# Comparative Analysis of Eleven Healthcare-Associated Outbreaks of Middle East Respiratory Syndrome Coronavirus (Mers-Cov) from 2015 to 2017

**DOI:** 10.1038/s41598-019-43586-9

**Published:** 2019-05-14

**Authors:** Sibylle Bernard-Stoecklin, Birgit Nikolay, Abdullah Assiri, Abdul Aziz Bin Saeed, Peter Karim Ben Embarek, Hassan El Bushra, Moran Ki, Mamunur Rahman Malik, Arnaud Fontanet, Simon Cauchemez, Maria D. Van Kerkhove

**Affiliations:** 1 Formerly Outbreak Investigation Task Force, Centre for Global Health, Institut Pasteur, 75015 Paris, France; 2 Present Address: Direction of infectious diseases, Santé publique France, Saint-Maurice, 94410 France; 3 Mathematical Modelling of Infectious Diseases, Institut Pasteur, UMR2000, CNRS, 75015 Paris, France; 4 Ministry of Health, Riyadh, Saudi Arabia; 5 Formerly Ministry of Health, Riyadh, Saudi Arabia; 6 Present Address: Department of Family and Community Medicine, College of Medicine, King Saud University, Riyadh, Saudi Arabia; 7 International Food Safety Authorities Network (INFOSAN) Management, Department of Food Safety and Zoonoses, World Health Organization, Geneva, Switzerland; 8 Department of Cancer Control and Policy, Graduate School of Cancer Science and Policy, National Cancer Center, Goyang, Korea; 9 Infectious Hazard Management Unit, Department of Health Emergencies, World Health Organization Regional Office for the Eastern Mediterranean, Cairo, Egypt; 10 Emerging Diseases Epidemiology Unit, Institut Pasteur, 75015 Paris, France; 11 Centre for Global Health, Institut Pasteur, 75015 Paris, France; 12 Conservatoire National des Arts et Métiers, Paris, France; 13 Infectious Hazards Management, Health Emergencies Programme, World Health Organization, Geneva, Switzerland

**Keywords:** Infectious diseases, Risk factors

## Abstract

Since its emergence in 2012, 2,260 cases and 803 deaths due to Middle East respiratory syndrome coronavirus (MERS-CoV) have been reported to the World Health Organization. Most cases were due to transmission in healthcare settings, sometimes causing large outbreaks. We analyzed epidemiologic and clinical data of laboratory-confirmed MERS-CoV cases from eleven healthcare-associated outbreaks in the Kingdom of Saudi Arabia and the Republic of Korea between 2015–2017. We quantified key epidemiological differences between outbreaks. Twenty-five percent (n = 105/422) of MERS cases who acquired infection in a hospital setting were healthcare personnel. In multivariate analyses, age ≥65 (OR 4.8, 95%CI: 2.6–8.7) and the presence of underlying comorbidities (OR: 2.7, 95% CI: 1.3–5.7) were associated with increased mortality whereas working as healthcare personnel was protective (OR 0.07, 95% CI: 0.01–0.34). At the start of these outbreaks, the reproduction number ranged from 1.0 to 5.7; it dropped below 1 within 2 to 6 weeks. This study provides a comprehensive characterization of MERS HCA-outbreaks. Our results highlight heterogeneities in the epidemiological profile of healthcare-associated outbreaks. The limitations of our study stress the urgent need for standardized data collection for high-threat respiratory pathogens, such as MERS-CoV.

## Introduction

**Figure 1:**
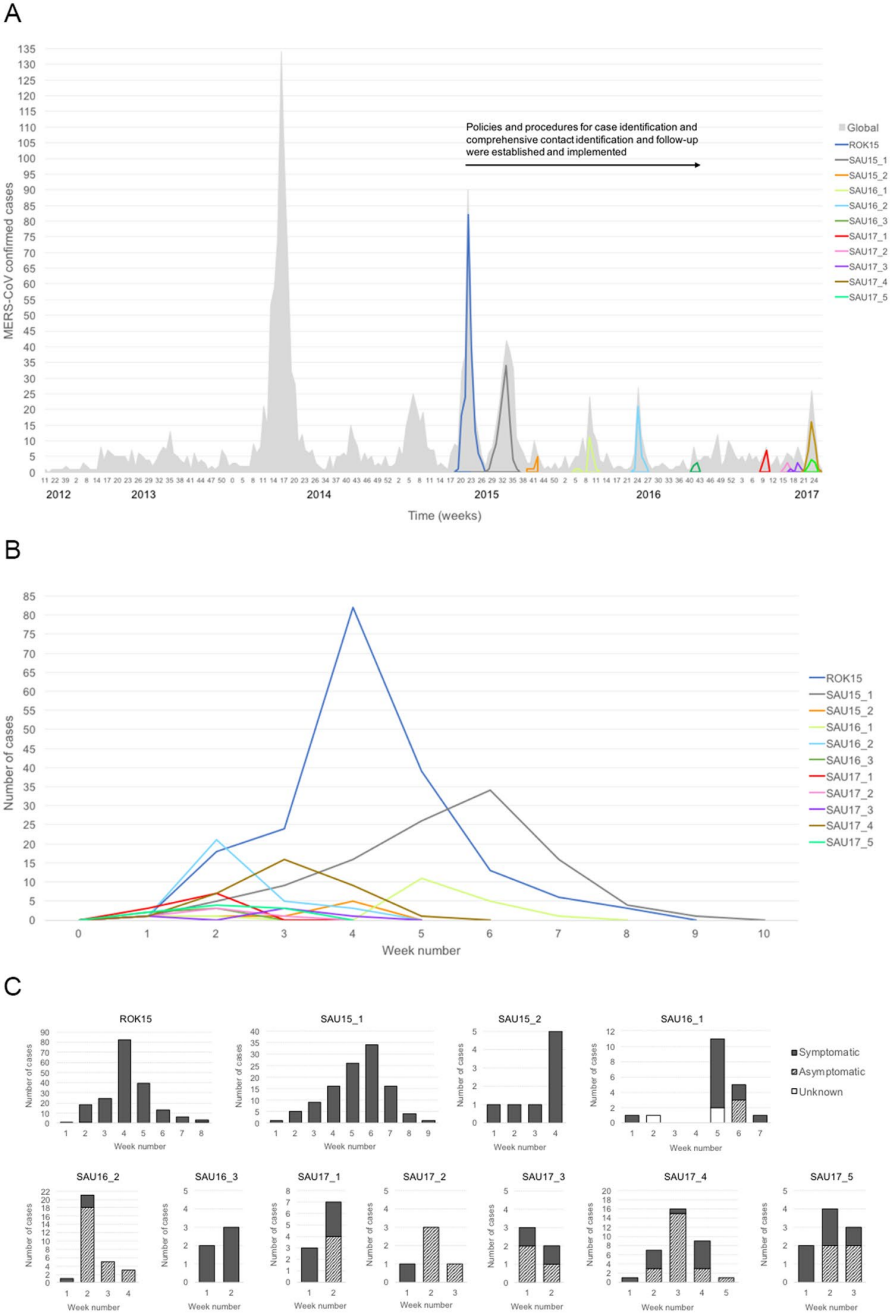
Epidemiological curves of MERS-CoV infections by outbreak. (**A**) Global MERS-CoV epidemiological curve. Gray surface: total weekly number of laboratory-confirmed MERS-CoV infections reported to WHO. Colored curves: HCA-outbreaks included in the study after systematic policies and procedures for case identification and comprehensive contact identification and follow up were established and implemented. (**B**) Weekly number of cases in each outbreak, each line representing an outbreak. Dark blue: ROK15; grey: SAU15_1; orange: SAU15_2; light green: SAU16_1; light blue: SAU16_2; dark green: SAU16_3; red: SAU17_1; pink: SAU17_2; purple: SAU17_3; brown: SAU17_4; turquoise: SAU17_5. (**C**) Epidemic curve for each HCA by week comparing symptomatic (dark grey), asymptomatic case (light grey) and unknown symptoms of laboratory confirmed cases (white). X axis represents the number of weeks since the first case was reported in each HCA-outbreak.

Since its emergence in 2012, the Middle East respiratory syndrome coronavirus (MERS-CoV) has caused recurrent spillovers from dromedary camel populations into the human population^1–3^. As of 1 October 2018, a total of 2260 laboratory-confirmed cases of MERS-CoV infection from 27 different countries, including 803 deaths, have been reported to the World Health Organization (WHO), with a large majority of them concentrated in the Arabian peninsula^4^. Although human-to-human transmission of MERS-CoV has so far been self-limiting^5–7^, substantial human-to-human transmission has been observed in healthcare settings^8–19^, reaching up to approximately 550 cases in six weeks in Jeddah and Riyadh in the spring 2014^20–22^ (Fig. 1A).

Such large healthcare-associated (HCA) outbreaks have mainly been limited to the Kingdom of Saudi Arabia (KSA) and the United Arabian Emirates (UAE) until the spring 2015, when a single imported case of MERS returning from the Middle East initiated a cluster of 186 cases in the Republic of Korea (ROK) across at least 17 hospitals and much of the country^18^. Super spreading events in healthcare settings has been described for several previous MERS outbreaks, including an outbreak in Al-Hasa governorate in 2013 and during the outbreak in ROK, where approximately 80% of the transmission events were epidemiologically linked to five MERS cases^14,18,23^. Superspreading events in health care facilities have been observed in similar high threat respiratory disease pathogens, such as Severe Acute Respiratory Syndrome (SARS) in Canada, China, Singapore^24–26^.

While more than half of the laboratory confirmed MERS-CoV infections reported globally to date are associated with human-to-human transmission in healthcare settings^27^, there has been little human-to-human transmission reported in household settings^28^. Outbreak investigations and scientific studies conducted during or after MERS hospital outbreaks have identified that aerosol-generating medical procedures with improper or inadequate personal protective equipment place medical personnel and patients sharing wards with MERS patients and family visitors at higher risk for MERS-CoV infection^29,30^, with exposure to infectious droplets being the likely source of contamination. Although close unprotected contact with a MERS patient is generally considered necessary for human-to-human transmission^31^, several studies have revealed that MERS-CoV particles can persist on surfaces as long as several days, raising the possibility of a role of fomites in transmission^32,33^. Fomite transmission is further supported by observed viral spreading between rooms that were clearly separated^15,18^ and outbreaks that occurred in hemodialysis units^14,15^.

Factors leading to healthcare-associated outbreaks include overcrowding in emergency departments, slow triage and isolation of suspected patients and inadequate compliance to infection prevention and control procedures^17,23,34^. However, few studies have described or compared the characteristics of HCA-outbreaks as a whole in terms of their size, epidemiologic factors^34,35^, or the role of interventions to stop transmission^23,36^. Here, we provide the largest comprehensive study of eleven healthcare-associated outbreaks that occurred between 2015 and June 2017. We carried out a comparative analysis of these outbreaks in terms of epidemiological profiles, demographic characteristics and clinical outcome.

## Methods

### Study design

We analyzed epidemiological datasets of laboratory-confirmed MERS patients and focused our study on eleven healthcare-associated outbreaks that were reported in KSA and ROK since 2015, when policies and procedures for case identification and comprehensive contact identification and follow up became systematic and were implemented by affected countries. The data used documented MERS-CoV infections reported to WHO under the International Health Regulations (2005). We only included clusters of cases/outbreaks that were linked to healthcare facilities. Supplemental ROK case-based data were provided as a detailed line list of the Korean MERS cases included in a published study^17^. We defined laboratory-confirmed MERS-CoV infection as following WHO guidelines^4,37^.

We defined a HCA-outbreak as the occurrence of 5 or more laboratory-confirmed MERS-CoV infections with reported epidemiologic links between cases and during which the human-to-human transmission events were documented within a single healthcare facility, with no more than 14 days apart between cases symptom onset. The MERS outbreak in the Republic of Korea in 2015 is treated as a single outbreak.

Individual-level variables included information on age, sex, nationality, occupation (healthcare personnel (HCP) yes/no), dates of symptom onset, date of notification to WHO, presence of any pre-existing co-morbid conditions, and clinical outcome. In case of missing or conflicting information and when information from the country was not available, we considered the data as missing.

### Statistical analysis

Descriptive analysis was performed by HCA-outbreak (outbreak-level analysis) using aggregated data, and for all cases (individual-level analysis). All analyses were conducted using Stata, version 14 (College Station, TX: StataCorp LP), Microsoft Excel (Version 15.35 2017, Jones, Chicago USA) and *R*.

#### Outbreak-level analysis

We calculated the duration, size and case fatality ratio for each outbreak. The duration of an outbreak was calculated as the number of days between the date of symptom onset of the first reported case to the date of symptom onset of the last reported case.

We obtained weekly smoothed estimates of the case reproduction number based on the approach developed by Wallinga and Teunis^38,39^ using the *R*_0_ package. We assumed that the serial interval of MERS-CoV had a Gamma distribution with a mean of 6.8 days and a standard deviation of 4.1 days, as described elsewhere^40^.

#### Individual-level analysis

We summarized case characteristics as frequencies and proportions for categorical variables, as median and interquartile ranges (IQR) for continuous variables. Chi-square tests were used to compare subgroups of cases when appropriate. A P value of less than 0.05 was used to indicate statistical significance. Univariate analysis identified variables significantly associated with fatal outcome, which were included in a multivariable model. Model selection was performed using a multilevel mixed-effects logistic regression with backwards selection taking into account clustering of individuals by outbreak. For the variable “age”, the cut-off was fixed at 65, based on the results of the univariate analysis. Variables with p-values < 0.05 were retained in the final model.

### Ethics

All data used in these secondary analyses were de-identified data obtained from WHO or datasets from peer-reviewed literature. As such, these data were deemed exempt from institutional review board assessment.

## Results

### General characteristics of HCA-outbreaks

Since 1 January 2015 to 1 October 2018, 2,260 laboratory-confirmed MERS-CoV infections have been reported to WHO. Figure 1A illustrates the global epidemic curve since MERS was first identified in humans. Each peak is associated with a health care associated outbreak (colored lines, Fig. 1A). From 2015, affected countries implemented systematic contact tracing and follow up (including laboratory testing), investigation and data collection of MERS suspect cases^41,42^.

In our analysis, a total of 423 laboratory-confirmed MERS cases from eleven distinct HCA-outbreaks during 2015–2017 were included (Table 1). The eleven HCA-outbreaks varied in terms of duration, size and epidemiological profile (Table 1, Fig. 1B). The median number of total reported cases per outbreak was 10 (interquartile range, [IQR] 6–27), ranging from 5 to 186 cases. The median duration was 20 days (IQR 16–23), ranging from 10 to 57 days.

**Table 1:** Characteristics of HCA MERS outbreaks from 2015–2017.

Outbreak	Country/City	Year of outbreak	Period of time*	Number of cases	Duration (days)	Initial R(t), median (95% CI)	Time to peak (weeks)	Delay onset to notification	Case fatality ratio	Age, median (IQR)	Male, n (%)	Asymptomatic, n (%)	Presence of comorbidity, n (%)	HCP, n (%)
ROK15	Republic of Korea	2015	11/05/15–03/07/15	186	53	5.7 (3.0–9.0)	4	6 (3–9)	18	55 (42–66)	110 (59)	1 (1)	83 (45)	32 (17)
SAU15_1	Riyadh	2015	13/07/15–08/09/15	112	57	2.9 (2.0–5.0)	6	5 (4–8)	50	58 (42–72)	68 (61)	0	82 (80)	15 (13)
SAU15_2	Al Manea	2015	03/10/15–22/10/15	8	19	1.4 (0.5–3.0)	4	8.5 (5–11.5)	75	57.5 (36.5–71)	6 (75)	0	6 (75)	2 (25)
SAU16_1	Buraidah	2016	06/02/16–13/03/16	19	36	1.0 (0.7–1.3)	4	4 (3–8)	42	36 (26–60)	15 (79)	3 (19)	11***	6 (32)
SAU16_2	Riyadh	2016	09/06/16–29/06/16	30	20	4.9 (2.7–7.3)	2	3 (3–4.5)	3	44.5 (32–58)	5 (17)	26 (87)	5***	17 (57)
SAU16_3	Hofouf	2016	10/10/16–20/10/16	5	10	1.6 (0.5–3.0)	2	3 (2–5)	40	55 (40–61)	4 (80)	0	3 (60)	2 (40)
SAU17_1	Wadi Aldwasser	2017	26/02/17–11/03/17	10	13	2.0 (0.3–4.3)	2	2.5 (2–5)	0	39 (32–52)	4 (40)	4 (40)	7 (78)	2 (20)
SAU17_2	Wadi Aldwasser	2017	11/04/17–26/04/17	5	15	3.0 (3.0–4.0)	2	2**	20	50 (31–55)	5 (100)	4 (80)	1***	1 (20)
SAU17_3	Riyadh	2017	24/04/17–15/05/17	5	21	1.0 (0.7–1.3)	3	2 (2–6)	20	33 (30–38)	3 (60)	3 (60)	1***	3 (60)
SAU17_4	Riyadh	2017	26/05/17–19/06/17	34	24	4.3 (1.5–7.5)	3	2 (2–3.5)	21	34.5 (30–54)	20 (59)	22 (65)	14 (42)	17 (50)
SAU17_5	Riyadh	2017	28/05/17–17/06/17	9	20	2.3 (0.5–4.5)	2	4 (3–4)	11	45 (42–48)	3 (33)	4 (44)	1 (11)	8 (89)

*Dates of symptom onset (or notification to WHO if the latter was not reported/available) of the first and the last cases. **No median or quartiles available: 4 cases out of 5 were notified to WHO the same day as the onset of symptoms. ***High proportion of missing values.

Three outbreaks began with sporadic cases during the first two to five weeks of the outbreak, while the other eight displayed a rapid increase to the peak. The median time between onset of symptoms of the first reported case and the peak of incidence was 3 weeks (IQR 2–3.75), ranging from 2 to 6 weeks.

The case fatality ratio (CFR) in outbreaks was 28% (116 reported deaths among 423 cases), compared with the global overall CFR of 35.5% (800 reported deaths among 2,254 cases reported as of 1 October^3^ (Table 1). During HCA outbreaks, CFR ranged from 0 to 75% (p < 0.01) and CFR was significantly lower among HCP MERS-CoV infections compared to non-HCP MERS-CoV infections (2% vs. 36% p < 0.01).

### Demographic and clinical characteristics

The demographic and clinical characteristics of the cases from HCA outbreaks included in our analyses are summarized in Table 1. The median age was 54 (IQR, 36–65), and significantly varied by outbreak (p < 0.001). Five outbreaks had a median age <40 and the other 6 outbreaks had a median age ≥50.

The majority of cases were male (57%, n = 243/423), and the sex ratio among cases differed significantly between outbreaks (p < 0.001). The overall proportion of HCP was 25% (n = 105/422). This proportion varied significantly by outbreak (p < 0.001), from 13% to 89% (Table 1). Median age was significantly lower among HCP than non-HCP cases (35 IQR 29–46 vs 58 IQR 45–70, p < 0.001) and the proportion of females was higher among HCP than non-HCP (70% vs 33%, n = 422, p < 0.001).

More than half (57%, n = 214/377) of cases had at least one underlying co-morbid condition (Table 1), and this was significantly lower among females compared to males (46% vs 64%, respectively, n = 377, p < 0.001) and among HCP compared to non-HCP (13% vs 70%, n = 376, p < 0.001).

Sixteen percent (n = 67/419) of cases were asymptomatic at time of reporting (Table 1). This proportion varied significantly between outbreaks ranging from 0% to 87% (n = 419, p < 0.001, Fig. 1C). Median age of asymptomatic cases was 34 (IQR, 30–48), the majority of whom were females (70%, n = 47/67) and had no underlying co-morbid conditions (78%, n = 29/37). The proportion of HCP among asymptomatic infections was high (70%, n = 47/67), and the CFR was null.

The median duration between symptom onset and case notification to WHO was 5 days (IQR 3–8).

### Risk factors associated with fatal outcome

In univariate analysis, fatal outcome was significantly associated with age (p < 0.001), presence of underlying comorbidities (p < 0.001), non-HCP status (p < 0.001), and male sex (p < 0.001, Table 2).

**Table 2:** Risk factors associated with the disease outcome among MERS cases (n = 423) identified in 11 HCA-outbreaks from 2015–2017.

Variables	Univariate Analyses			Multivariate Analyses		
	OR	p-value	95%CI	Adjusted OR	p-value	95%CI
Age						
<65	1			1		
≧65	7.50	<0.001	4.39–12.77	4.79	<0.001	2.60–8.64
Underlying medical condition (yes vs. no)	10.12	<0.001	5.07–20.21	2.74	0.007	1.32–5.70
Health care personnel status (HCP vs. non-HCP)	0.03	<0.001	0.01–0.15	0.07	0.001	0.01–0.35
Gender (male vs. female	2.74	<0.001	1.69–4.44	—	—	—

OR: odds ratio. Adj. OR: adjusted odds ratio. Analysis using individual-level data. Univariate comparison of the association between the probability of fatal outcome and each categorical variable, using the chi-square test with a significance threshold at 0.05. Multilevel mixed-effects logistic regression model with a random effect (outbreak) and adjusting for potential confounding factors, with an exclusion threshold of 0.05 (n = 376, p < 0.001). Missing values were excluded from both analyses.

In multivariate analysis, patients ≥65 years old (OR 4.79, 95% CI: 2.60–8.64) and the presence of ≥1 underlying comorbid condition (OR 2.74, 95% CI: 1.32–5.70) had an increased risk of death. HCP status was associated with a decreased risk of death (OR 0.07, 95% CI: 0.001–0.35) (Table 2).

### Estimation of time-varying reproduction number

**Figure 2:**
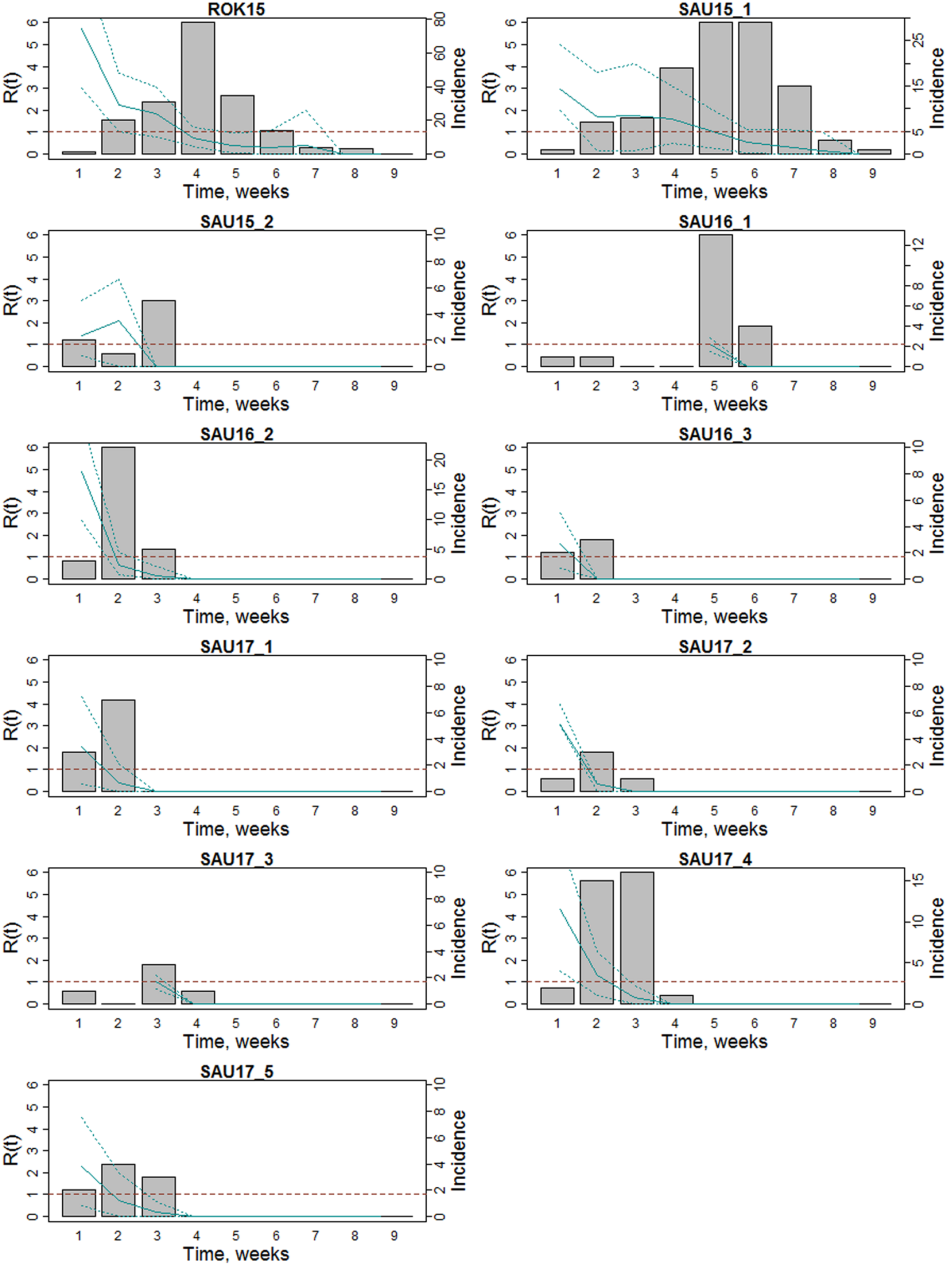
Weekly estimates of the case reproduction number R_(t)_ for 11 HCA-outbreaks between 2015 and 2017. Weekly R_(t)_ estimates per outbreak are shown (plain blue line) with their 95% confidence intervals interval (dotted blue lines) (left Y axis). The bar chart represents the weekly incidence (right Y axis). The horizontal dotted red line represents the R(t) threshold set at 1.

At the start of each HCA outbreaks, the case reproduction number *R*_*(t)*_ ranged from 1.0 (95% CI 0.7–1.3) to 5.7 (95% CI 3.0–9.0) (Table 1 and Fig. 2). Estimates of *R*_*(t)*_ dropped below 1 within 2 to 6 weeks from the first reported case in the outbreak (n = 11 outbreaks, median 3 weeks, IQR 2–4).

## Discussion

We provide here a comparative characterization of MERS HCA-outbreaks and report substantial heterogeneity between HCA-outbreaks illustrating the complexity of the factors contributing to the emergence of a cluster of cases associated with nosocomial transmission.

The duration and epidemic profiles of outbreaks varied; some started with an apparent sharp increase in incidence while others began more slowly with isolated cases emerging intermittently for a few weeks before a cluster of cases appeared in a healthcare facility. Some outbreaks had a sharp decline in cases, while others experienced a long tail lasting several weeks after the peak.

The median estimates of the reproduction number *R*(t) in the early stages of outbreaks included in our analyses reached as high as 5.7 in the Republic of Korea, as has been found by others^43^, likely facilitated by multiple superspreading events at two hospitals^43^. What is perhaps most informative from a public health perspective is the length of time it took to bring the outbreaks under control. All of outbreaks reached *R*_*t*_ values below 1 within 2 to 6 weeks after the first cases were identified, highlighting that the time frame in which hospital and ministry officials can implement control measures to stop nosocomial outbreaks.

Factors explaining differences in HCA outbreak size and duration might include variations in the speed in which cases were suspected and timing of interventions implemented in healthcare settings, including contact identification, management and isolation of patients, improved infection prevention and control measures and in some cases, the requirement to close departments^14–18,29^. In this study, we were not able to evaluate the impact of interventions in these outbreaks.

Prevention of large HCA outbreaks since 2014 (Fig. 1A), may be, in part, explained by improvements in contact tracing policies implemented in 2015. In 2015, contact tracing became more systematic with the identification and follow up of high (close, unprotected contact) and low risk contacts (protected HCW). In affected countries, National Ministries of Health and hospital staff comprehensively list all contacts of known MERS patients, including healthcare workers at all facilities/departments the patient visited, patients who shared wards/rooms with MERS patients, family and visitors and occupational contacts. Follow up of contacts includes the testing of all high-risk contacts, regardless of the development of symptoms. Recommendations stated that positive contacts are placed in quarantine (home or hospital isolation for asymptomatic or symptomatic secondary cases, respectively) until they test negative^41,44–46^. Additionally, affected countries enhanced infection prevention and control procedures education, and training, and implemented visual triage systems^41^ to reduce delays in testing, isolation and care of suspected MERS-CoV patients. This has again been recently illustrated by the lack of secondary cases following the identification of a confirmed case of MERS in Korea in September 2018^47^ was due to the rapid and comprehensive isolation, treatment and management of contacts of the patient.

The variation in outbreak size and duration is also affected by superspreading events early in some outbreaks, during which a limited number of cases generated a disproportionately large proportion of the secondary cases under specific conditions in hospitals, occurring in some outbreaks^48–50^. Two super spreading events have been documented in KSA and in the Republic of Korea. In the Republic of Korea, the practice of “doctor shopping”, extended stays in overcrowded emergency departments, cultural practices of large numbers of family members visiting sick relatives, and environmental contamination amplified transmission from some patients to others^14,17,18,51^. During the outbreak in KSA in 2015 at the Ministry of the National Guard Hospital, a high number of secondary cases were among HCP very quickly after the hospitalization and a surgical procedure of the index case^16^. These events triggered comprehensive review IPC in hospitals, emergency department layout, movements of patients, triage of respiratory visits, duration of emergency department stay, training of hospital staff and disinfection of healthcare facilities.

Our study confirmed that age and presence of comorbidities are linked to increased risk of death, similar to previously published results^52,53^ whereas being HCP was protective. The protective effect of HCP could be explained by the fact that HCP are more likely to be younger (<60 years old) and have fewer underlying medical conditions than hospitalized patients, but also that they are likely to be identified earlier or seek medical care soon following contact with a confirmed patient.

The proportion of asymptomatic secondary cases identified during outbreaks has increased since 2014. There is no evidence to suggest that this represents changes in virus pathogenicity, epidemiology or transmission patterns of MERS in recent years. However, the increase in the number of reported asymptomatic cases is hypothesized to be due to earlier detection efforts from more aggressive contact identification and testing during HCA-outbreaks since 2015 as testing policies adopted and implemented by KSA and other countries have changed following the large outbreaks in Jeddah/Riyadh in 2014^3,41,54^. In 2017, 40–80% of the laboratory confirmed HCP secondary cases experienced no symptoms and were detected as part of a policy to test all contacts irrespective of symptoms (Table 1). We believe that the identification of HCP asymptomatic cases, and their subsequent isolation, has had a strong impact on prevent further human to human transmission in health care settings. This is visually demonstrated in Fig. 1C by the outbreak labelled SAU16_2, which included 26 (of 30 reported cases) asymptomatic cases. While this is a large number of secondary cases, we argue that the early identification, isolation and recovery of these asymptomatic/mildly symptomatic cases effectively stopped human to human transmission.

Our study has several limitations due to variability in the completeness and quality of case-based data provided to WHO since 2012 and also due to the lack of detailed information on the timing specific interventions were implemented in relation to each outbreak. Without detailed information on the timing of interventions in each health care facility it was not possible in our analyses to determine which intervention or combination of interventions had the greatest impact on stopping the MERS outbreaks. Moreover, prior to 2015, contacts without symptoms were not tested for MERS-CoV infection, thus the rate of identification of secondary cases was drastically different prior to 2015, which complicates the comparison of data collected before and after 2015. The improvements in data reporting on cases (e.g., more systematic reporting of underlying conditions, reported exposures, contacts between patients) from 2015 allowed us to perform better analyses with less missing values.

We continue to encourage the policy of identifying, following and testing of all high risk contacts of MERS patients in HCA-outbreaks^3,41,55^. The natural history of asymptomatic infection and role of asymptomatic or mildly symptomatic HCP in transmission of the virus between patients, requires detailed scientific studies to better understand their potential role in transmission^15^.

The sharing of outbreak experiences between affected hospitals within and between countries and a detailed evaluation of the impact of non-therapeutic interventions is critical to our understanding and for the prevention of nosocomial outbreaks of respiratory pathogens. Health care professionals and hospitals currently have tools to limit the extent and impact of such events, which include early identification and isolation of suspect patients and strict adherence to standard infection prevention and control measures. These are the hallmark of effective MERS-CoV control. A combination of interventions including the efficient triage of patients with respiratory symptoms at hospital entry; limiting wait times and overcrowding in waiting areas; isolation of suspected and confirmed cases; appropriate use of droplet personal protection equipment by HCP; basic hand hygiene; increased protective aerosol precautions for HCP during aerosol-generating medical procedures; efficient surface and environmental decontamination of areas with MERS patients, and extensive contact tracing, can prevent human to human transmission in health care settings.
